# Fucosyltransferase 1 and 2 play pivotal roles in breast cancer cells

**DOI:** 10.1038/s41420-019-0145-y

**Published:** 2019-03-06

**Authors:** Tai-Yu Lai, I-Ju Chen, Ruey-Jen Lin, Guo-Shiou Liao, Hui-Ling Yeo, Ching-Liang Ho, Jen-Chine Wu, Nai-Chuan Chang, Andy Chi-Lung Lee, Alice L. Yu

**Affiliations:** 1 Institute of Stem cell and Translational Cancer Research, Chang Gung Memorial Hospital, Linkou, Taiwan; 2 Genomics Research Center, Academia Sinica, Taipei, Taiwan; 3 Tri-service General Hospital, Department of Surgery, National Defense Medical Center, Taipei, Taiwan; 4 Department of Pediatrics, University of California in San Diego, San Diego, USA

## Abstract

FUT1 and FUT2 encode alpha 1, 2-fucosyltransferases which catalyze the addition of alpha 1, 2-linked fucose to glycans. Glycan products of FUT1 and FUT2, such as Globo H and Lewis Y, are highly expressed on malignant tissues, including breast cancer. Herein, we investigated the roles of FUT1 and FUT2 in breast cancer. Silencing of FUT1 or FUT2 by shRNAs inhibited cell proliferation in vitro and tumorigenicity in mice. This was associated with diminished properties of cancer stem cell (CSC), including mammosphere formation and CSC marker both in vitro and in xenografts. Silencing of FUT2, but not FUT1, significantly changed the cuboidal morphology to dense clusters of small and round cells with reduced adhesion to polystyrene and extracellular matrix, including laminin, fibronectin and collagen. Silencing of FUT1 or FUT2 suppressed cell migration in wound healing assay, whereas FUT1 and FUT2 overexpression increased cell migration and invasion in vitro and metastasis of breast cancer in vivo. A decrease in mesenchymal like markers such as fibronectin, vimentin, and twist, along with increased epithelial like marker, E-cadherin, was observed upon FUT1/2 knockdown, while the opposite was noted by overexpression of FUT1 or FUT2. As expected, FUT1 or FUT2 knockdown reduced Globo H, whereas FUT1 or FUT2 overexpression showed contrary effects. Exogenous addition of Globo H-ceramide reversed the suppression of cell migration by FUT1 knockdown but not the inhibition of cell adhesion by FUT2 silencing, suggesting that at least part of the effects of FUT1/2 knockdown were mediated by Globo H. Our results imply that FUT1 and FUT2 play important roles in regulating growth, adhesion, migration and CSC properties of breast cancer, and may serve as therapeutic targets for breast cancer.

## Introduction

Glycoconjugates have long been recognized as essential components of many living organisms. A number of studies have documented the roles of glycoconjugates in a variety of diseases such as viral and bacterial infection, inflammation, autoimmune dysfunction, or cancer metastasis^1^. However, our knowledge on how glycoconjugates are involved in these processes remains limited. Recently, specific glycan structures have been reported to correlate with breast tumor progression, such as sialyl-Tn (sTn), Lewis^y^ (Le^y^), sialyl-Lewis^a^ (sLe^a^), sialyl-Lewis^x^ (sLe^x^), sLe^x^-Le^x^, Thomas Friedrich (TF), Globo H, polysialic acid (PSA) and GM2^2–5^.

Among these tumor associated glycans, the terminal alpha 1, 2-linked fucose of Lewis^y^ and Globo H are synthesized by alpha 1, 2 fucosyltransferase, FUT1 and FUT2, in human^6,7^. These alpha 1, 2 fucosyltransferases are Golgi stack membrane enzymes that catalyze the transfer of alpha 1, 2-linked fucose to the galactose residue of glycans. In addition to breast cancer, altered cell surface alpha 1, 2-fucosylated glycans have been found in a variety of malignancies such as cancers of colon, pancreas, endometrium, cervix, bladder, lung and choriocarcinoma.

FUT1 and FUT2 null mice develop normally and exhibit no gross phenotypic abnormalities, despite absence of Fucα2Galβ epitope in the epididymal epithelium and uterine epithelium, respectively^8^. It has also been shown that FUT1 and FUT2 selectively substitutes galactose residue on glycoconjugates in tissue-specific manner. For example, fucosyl GA1 (FGA1) is lost from pancreas in FUT1-null mice, whereas, both FGA1 and fucosyl GM1 (FGM1) are completely absent in antrum, cecum, and colon in FUT2-null mice^9^. In addition, FUT1 and FUT2 are capable of generating FGM1 and Globo H in small cell lung cancer cells and breast cancer cells, respectively^10,11^. A numbers of recent studies have implicated important functions of FUT1 and FUT2 in colon cancers. For instance, FUT1 overexpression in HT-29/M3 colon cancer cells induces synthesis of H type 2 and Le^y^ and decrease in sLe^x^, which results in altered glycosylation patterns of MUC1 and MUC5AC apomucins with reduced interaction with E-selectin, leading to greater invasive and metastatic capacities^12–14^. Indeed, overexpression of FUT1 in rat colon carcinoma results in increased tumorigenicity, increased resistance to apoptosis and facilitated escape from immune surveillance^15,16^. In addition to colon cancer, FUT1 transgenic studies show enhanced vasculogenesis and gastrointestinal metastatic ability of pancreatic cancer cells (BxPC3), but greatly retarded the growth of hepatic cancer cells (HepG2) due to dramatic decrease in sLe^x^ expression, increase in Le^y^ and Le^b^ expression with failure to interact with endothelial E-selectin^14,17,18^. Suppression of FUT1 and FUT4 by siRNA reduces Le^y^ expression and inhibits cell proliferation through decreased EGFR signaling pathway in epidermoid carcinoma cells (A431)^19^. Recent studies have shown that alpha 1, 2 fucosyltransferase induces angiogenesis by activating ERK1/2, promotes metastasis by increasing MMP-2 and MMP-9, and accelerates hepatocellular carcinoma progression by influencing Notch signaling and multidrug resistance by inducing PI3K/Akt signaling pathway^20–23^.

In breast cancer, GSL profiling by mass spectrometry showed that FUT1 contributed to the biosynthesis of Globo H and fucosyl-lactoceramide^24^ with our previous report that both FUT1 and FUT2 contribute to the expression of Globo H in breast cancers^10^. We also demonstrated that FUT1 regulated fucosylation of LAMP-1 and LAMP-2 to modulate autophagic flux rate via mTOR signaling and autolysosome formation^25^. An in vitro study showed that FUT1 knockdown reduces cell proliferation of a HER2-overexpressing gastric cancer cells (NCI-N87)^26^. However, the roles of FUT1 and FUT2 in cancer stem cells (CSCs) and tumorigenicity of breast cancer have yet to be delineated.

Here, we investigated the impacts of alpha 1, 2-linked fucose on breast cancer biology and CSC properties using lentiviral system to knockdown and electroporation to overexpress FUT1 and FUT2.. We showed that FUT1 and FUT2 played pivotal roles in the regulation of CSC properties, including cell proliferation, epithelial-mesenchymal transition, tumorigenesis, and metastasis.

## Results

### Silencing of FUT1 and FUT2 inhibits cell proliferation in vitro and tumorigenicity in vivo

**Fig. 1:**
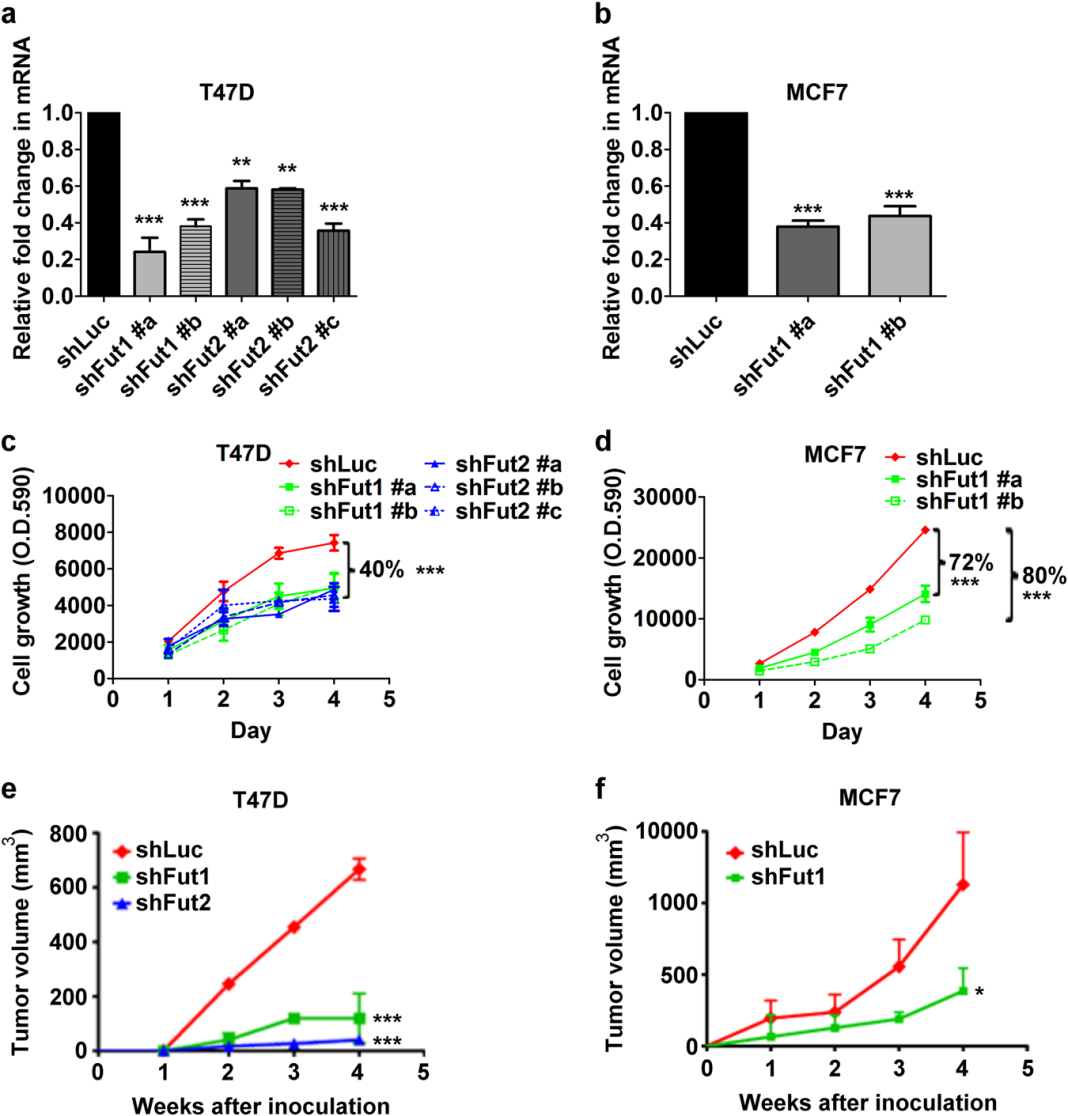
Silencing FUT1 or FUT2 decreases cellular growth of breast cancer cells and suppresses xenograft tumor growth. **a,** T47D or **b,** MCF7 cells stably expressing shLuc, shFUT1, or shFUT2 were analyzed for FUT1 or FUT2 mRNA expression. Different shRNA clones were designated as #a, #b, or #c. Cell proliferation of **c,** T47D or **d**, MCF7 cells expressing shLuc, shFUT1, or shFUT2 were determined by alamar blue assay. Tumor volume of **e**, T47D and **f**, MCF7 were generated with data from 5 mice in each group. Values are mean ± SEM from three independent experiments. *p < 0.05; **p < 0.01; ***p < 0.0001

To investigate whether inhibition of FUT1 or FUT2 has functional consequences in breast cancer, lentiviral-based short hairpin RNA (shRNA) was employed to establish stable cell lines with silenced expression of FUT1 (shFUT1) or FUT2 (shFUT2) in T47D cells, which expressed significantly more FUT2 than FUT1, and MCF7 cells, which expressed high FUT1 but negligible FUT2. As shown in Fig. 1a, transfection of T47D cells with two lentiviral constructs of shFUT1 (shFUT1 #a and shFUT1 #b) suppressed FUT1 expression to 24% and 38% of control shLuc, respectively, and transfection with three lentiviral constructs of shFUT2 (shFUT2 #a, shFUT2 #b, and shFUT2 #c) reduced FUT2 expression to 59%, 58%, and 35% of control shLuc. Similarly, FUT1 expression in MCF-7 cells was suppressed to 38% and 43% of control by shFUT1#a and shFUT1#b, respectively (Fig. 1b). As shown in Fig. 1c, d, the cell proliferation rates were reduced by FUT2- and FUT1-silencing in T47D to 60% of control cells expressing shLuc, and by shFUT1#a and #b in MCF7 cells to 20% and 28%, respectively. To further examine if this in vitro phenotype could be recapitulated in vivo, FUT1 or FUT2-silenced T47D and MCF7 cells were inoculated into the mammary fat pad of NOD/SCID mice. Tumor growth rates in T47D and MCF7 xenografts were significantly decreased by 65% and 70%, respectively, upon FUT1 knockdown (Fig. 1e, f). FUT2-silencing in T47D dramatically reduced tumor volume by 95% (Fig. 1e). In short, both FUT1 and FUT2 are important for tumor growth in vitro and in vivo.

### Silencing of FUT1 and FUT2 reduces mammosphere formation and CSC-like subpopulation

**Fig. 2:**
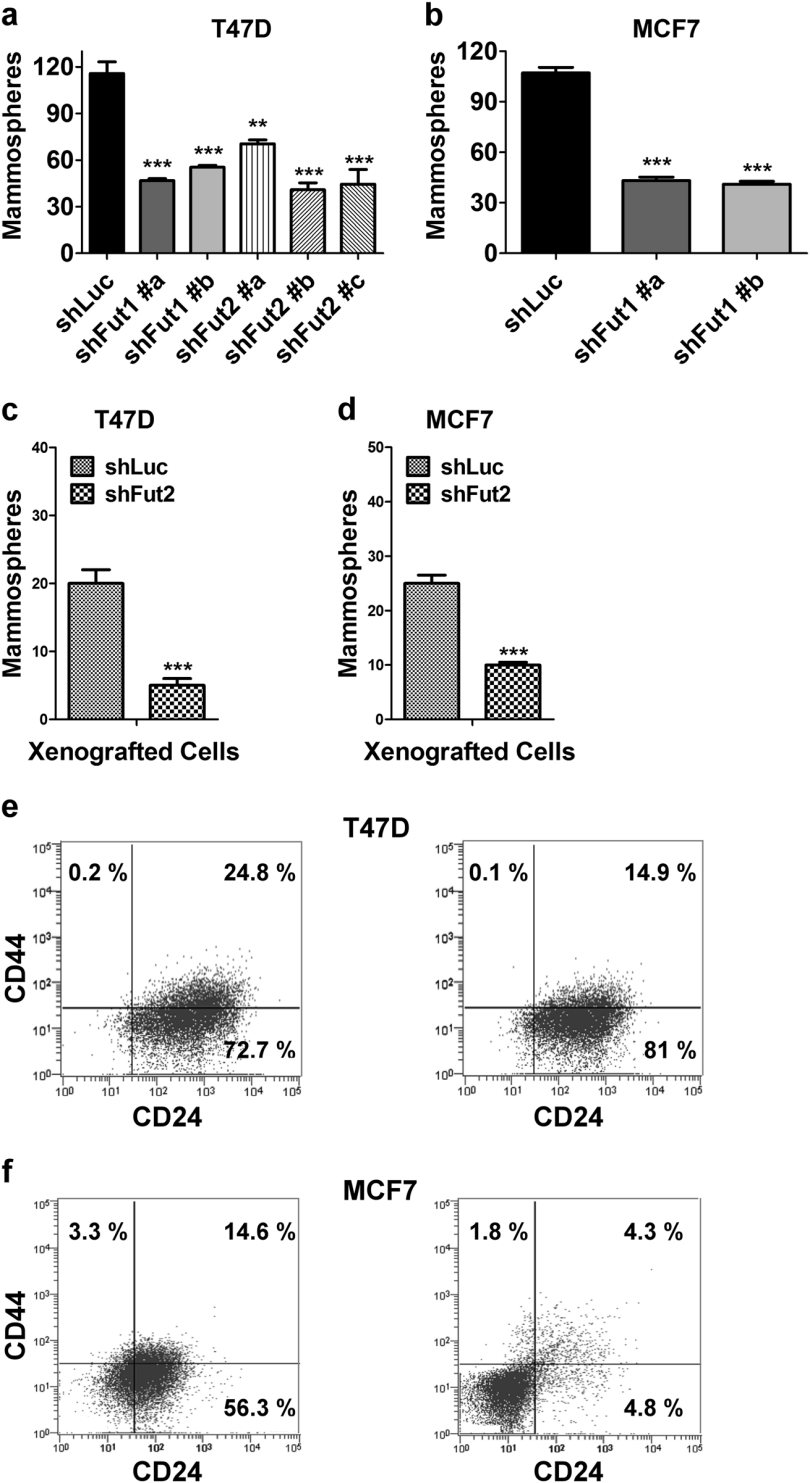
Silencing FUT1 or FUT2 reduces mammosphere formation in T47D or MCF7 cells. **a,** T47D or **b,** MCF7 cells stably expressing shLuc, shFUT1, or shFUT2 were analyzed for their mammosphere forming capability. **c**, **d,** T47D or MCF7 xenografted tumors with shLuc control, FUT1 knockdown, or FUT2 knockdown were harvested to determine their mammosphere forming capability. Values are mean ± SEM from three independent experiments. Representative flow analysis of CSC subpopulation in **e**, T47D or **f**, MCF7 cells expressing shLuc, shFUT1, or shFUT2. *p < 0.05; **p < 0.01; ***p < 0.0001

Accumulating evidence suggests that breast cancer is initiated and maintained by a small subpopulation of cancer stem cells that possess the capacity for self-renewal which drives tumorigenesis and aberrant differentiation that provides cellular heterogeneity^27,28^. Such breast cancer stem cells display the ability to form mammospheres in vitro^29^. FUT1 knockdown significantly reduced the capacity of mammosphere forming capacity of T47D and MCF7 cells to 40% of control cells expressing shLuc (Fig. 2a, b). The FUT2 silenced T47D only formed 35% mammosphere of control cells (Fig. 2a). In cells harvested from xenografted tumor of T47D and MCF7, silencing of FUT2 or FUT1 similarly reduced mammosphere formation by 75% and 65%, respectively, as compared to that in shLuc control (Fig. 2c, d). In addition, knockdown of FUT2 in T47D and FUT1 in MCF7 cells reduced the CSC-enriched subpopulation (CD44^+^/CD24^−^) from 0.2% to 0.1% and from 3.3% to 1.8%, respectively (Fig. 2e, f). The CD44^+^/CD24^+^ subpopulation also decreased from 24.8% to 14.9% in T47D and from 14.6% to 4.3% in MCF7 (Fig. 2e, f). According to the mainstream literature, CD44^+^/CD24^−^ has been designated as CSC marker of breast cancer, including T47D and MCF-7;^30,31^ however, a few other studies suggested CD44^+^/CD24^+^ subpopulation also display CSC-like properties^32^. These findings demonstrate that both FUT1 and FUT2 play important roles in the functional properties of breast cancer stem cells.

### Silencing of FUT2 reduces cell adhesion and cell-matrix interaction

**Fig. 3:**
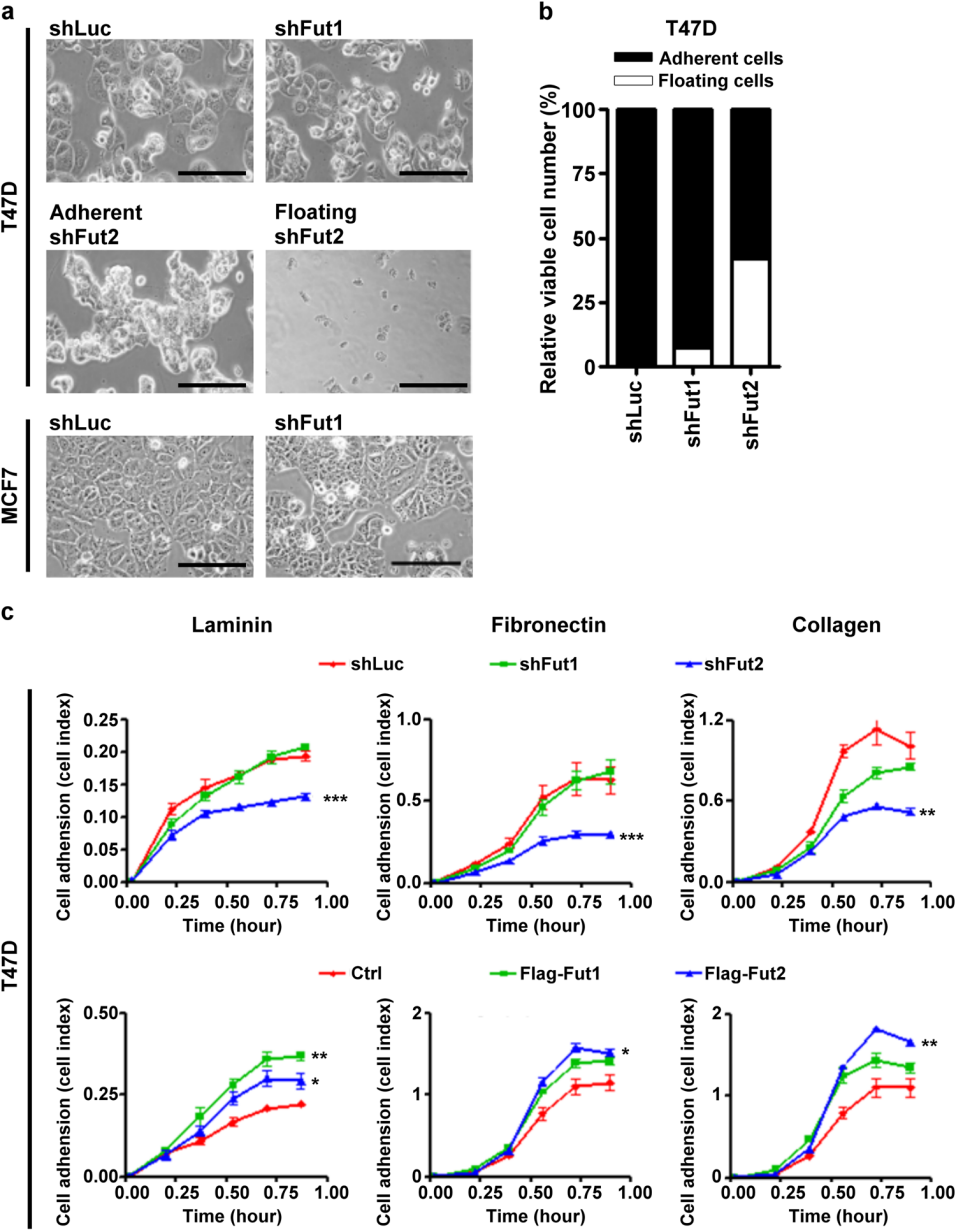
Silencing FUT2 influences cell morphology and adhesion ability. **a,** A large portion of T47D cells expressing shFUT2, but not T47D or MCF7 cells expressing shFUT1, detached from culture dish at 72 h after lentiviral infection. Scale bars, 200 μm. **b,** Adherent cells and floating cells were collected and analyzed for cell number and viability by cell viability analyzer. **c,** The adhesion ability of T47D cells expressing shLuc, shFUT1, or shFUT2 cultured on plate coated with laminin, fibronectin, or collagen were measured by RT-CES system up to 1 h. Values are mean ± SEM from three independent experiments. *p < 0.05; **p < 0.01; ***p < 0.0001

FUT2 knockdown changed the morphology of T47D from large cuboidal cells to dense clusters of small and round cells with increased number of detached floating cells (Fig. 3a), accounting for 42% of the cells (Fig. 3b), while silencing FUT1 in T47D had no significant effect on the cell morphology although the growth rate was retarded (Fig. 3a and 1c). MCF7 with shLuc control grew as monolayer of polygonal cells which were well spread out on culture dishes, whereas FUT1 knockdown displayed an unusual growth pattern with clusters of 15–30 small, round to oval cells surrounded by sharply demarcated borders (Fig. 3a). To determine whether cell-matrix interaction was affected by FUT1 and FUT2, cell adhesion assay was performed in 96-well microtiter plates coated with laminin, fibronectin, or collagen IV. FUT2 knockdown in T47D cells significantly decreased cell-matrix interactions, whereas FUT2 overexpression (Supplementary Figure 1a) increased cell adhesion to all 3 matrix proteins (Fig. 3c). In contrast, FUT1 knockdown had no effects on cell-matrix interaction except for a slightly reduced adhesion to collagen IV, while FUT1 overexpression enhanced the cell adhesion to laminin and collagen IV (Fig. 3c). These results indicate that FUT2 is a key regulatory of cell adhesion to extracellular matrix components.

### FUT1 and FUT2 regulate cell motility

**Fig. 4:**
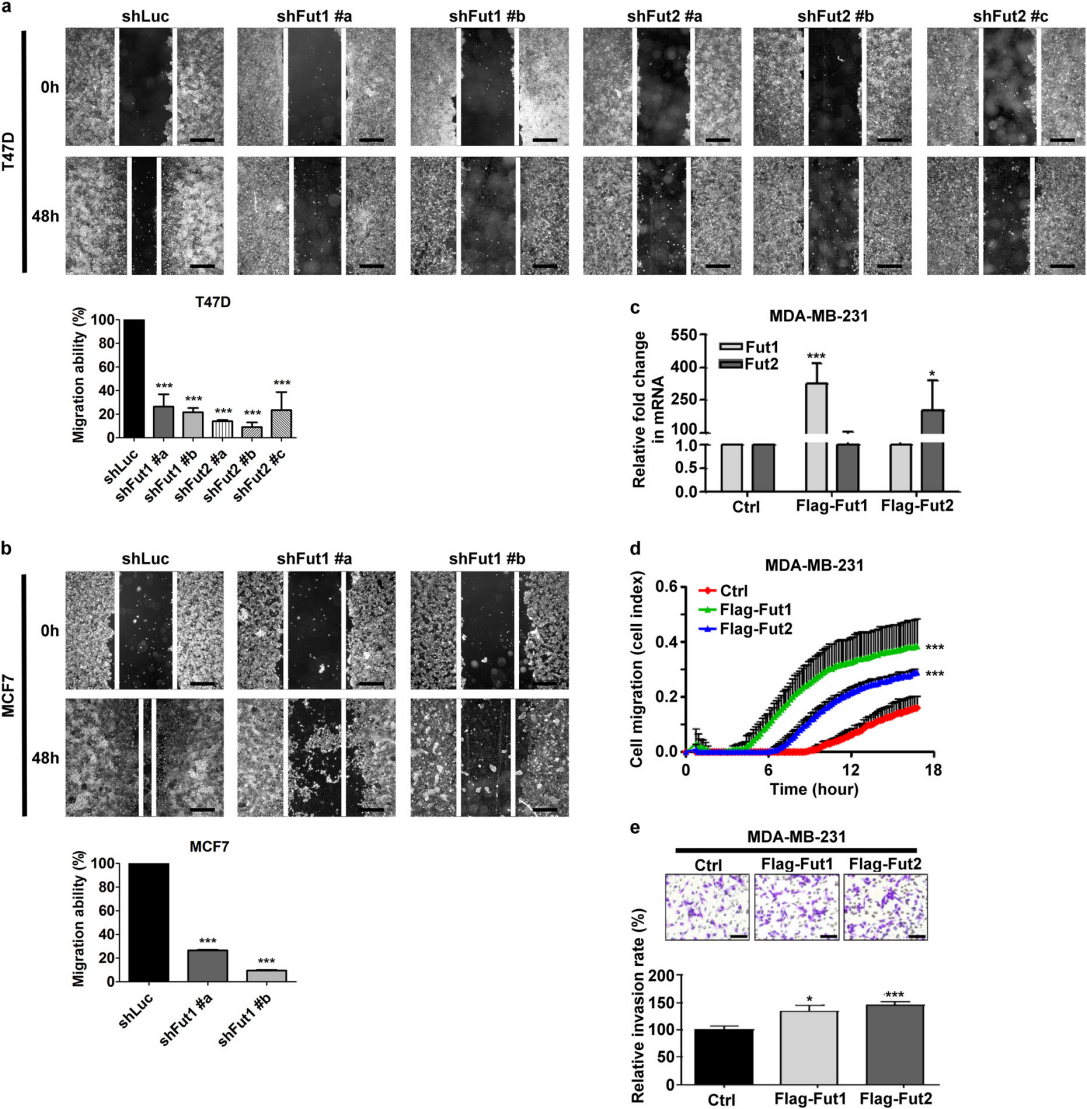
FUT1 and FUT2 are involved in cell migration. Cell motility of **a,** T47D or **b,** MCF7 cells expressing shLuc, shFUT1, or shFUT2 was analyzed by in vitro wound healing assay. The images were taken at 0 h and 48 h after the wounds were made. Scale bars, 500 μm. Bar graphs showed percentage of remaining wound area over that of cells at 0 h, and normalized to shLuc control, which was set as 100%. **c,** MDA-MB-231 cells expressing vector control, Flag-FUT1, or Flag-FUT2 were analyzed for FUT1 or FUT2 mRNA expression. **d,** Cell migration toward serum of MDA-MB-231 cells with indicated conditions was determined by a RT-CIM system. **e,** Transwell assay for cell invasion of MDA-MB-231 cells with indicated conditions. Values are mean ± SEM from three independent experiments. Scale bars, 50 μm. *p < 0.05; **p < 0.01; ***p < 0.0001

To further investigate whether FUT1/FUT2 play a role in cell migration, wound healing assay was performed. FUT1 and FUT2 knockdown significantly diminished the speed of wound closure to 20% and 15%, respectively, of control cells both in T47D and MCF7 (Fig. 4a, b). In addition, a highly-invasive breast cancer cell line, MDA-MB-231, was used to investigate cell migration and invasion toward serum. Both FUT1 and FUT2 overexpression (Fig. 4c) markedly enhanced the transwell migration rate by 3.33 fold and 2.22 fold, respectively, of cells expressing vector control (Fig. 4d). In the matrigel invasion assay, FUT1 and FUT2 overexpression also increased cell invasion rate by 1.33 fold and 1.45 fold of control, respectively (Fig. 4e). Furthermore, to determine whether FUT1 and FUT2 promote breast cancer metastasis in vivo, MDA-MB-231 stably and constitutively expressing luciferase by lentiviral-mediated gene transfer was transfected with empty vector, Flag-FUT1 or Flag-FUT2. After tail vein injection of these cells into NOD/SCID mice, FUT1 and FUT2 overexpression enhanced tumor localization in the lungs by 2.56 fold and 5.3 fold, respectively of vector control cells as determined by luciferase luminescence (Supplementary Figures 2a and b). This was consistent with greater tumor mass in lungs as reflected by weight in mice injected with FUT1 and FUT2 overexpressing cells than that in vector control cells (Supplementary Figures 2c and d). These results indicate that FUT1 and FUT2 are important players in wound healing, cell migration, and cell invasion.

### FUT1 and FUT2 regulate epithelial-mesenchymal transition (EMT)

**Fig. 5:**
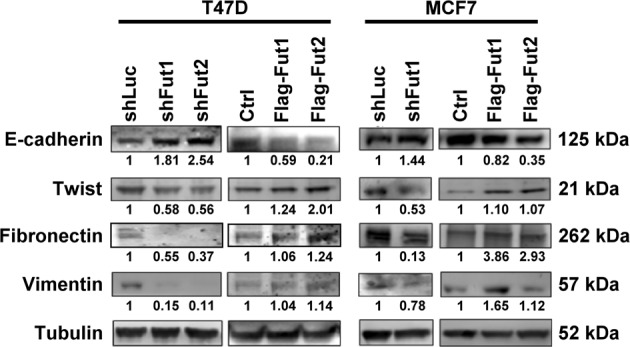
FUT1 and FUT2 regulate the changes of EMT markers. Western blots analysis showing expression of EMT markers in T47D or MCF7 cells with vector control, FUT1 or FUT2 overexpression, or, FUT1 or FUT2 knockdown

In light of the impact of FUT1 and FUT2 on cancer stem cells (CSC) and cell metastasis, we investigated the role of FUT1 and FUT2 in epithelial-mesenchymal transition (EMT), which was considered a hallmark of CSC and metastasis. The expression level of EMT markers was determined by western blotting. As shown in Fig. 5, both FUT1 and FUT2 knockdown enhanced levels of E-cadherin, while suppressing the levels of Twist, fibronectin and vimentin in both T47D and MCF7 cells. In contrast, FUT1 and FUT2 overexpression decreased levels of E-cadherin and increased levels of fibronectin in MCF7, and twist in T47D. These data support the involvement of FUT1 and FUT2 in EMT process.

### Contribution of Globo H-ceramide to FUT 1 and FUT2 regulated cell migration and adhesion

**Fig. 6:**
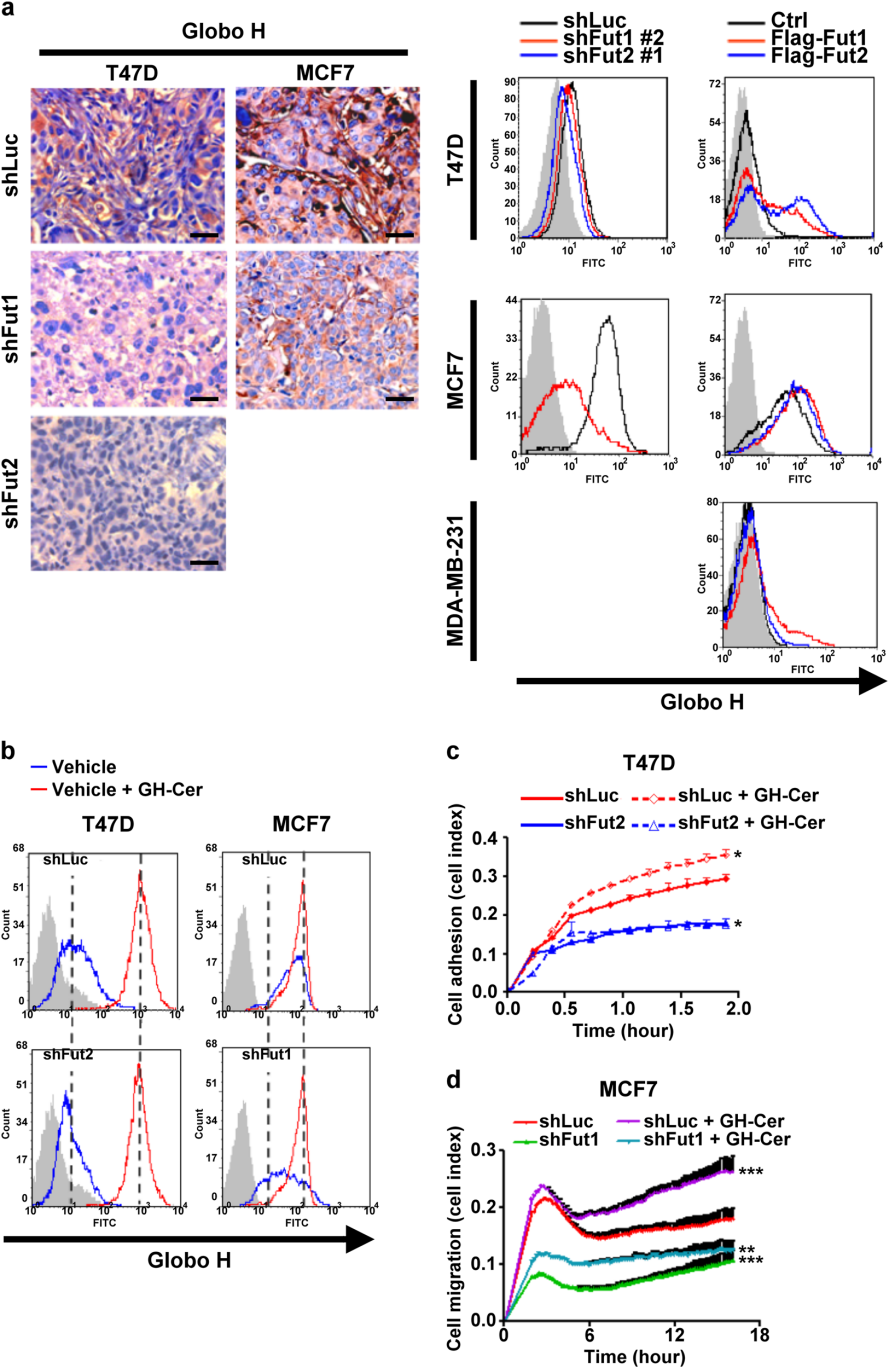
FUT1-modified glycans positively regulate cell migration through Globo H. **a,** Immunohistochemistry staining of Globo H expression in excised T47D or MCF7 tumor xenografts expressing shLuc, shFUT1, or shFUT2 and flow cytometry analysis of Globo H in T47D, MCF7, and MDA-MB-231 cells with indicated conditions. Scale bars, 50 μm. **b,** Flow analysis of Globo H expression in T47D or MCF7 cells expressing shLuc, shFUT1, or shFUT2 with or without Globo H-ceramide. **c,** Cell adhesion ability of T47D cells expressing shFUT2 or shLuc to polystyrene in the presence or absence of Globo H-ceramide were measured by RT-CES system up to 2 h. **d,** Cell migration toward serum of MCF7 cells expressing shLuc or shFUT1 in the presence or absence of Globo H-ceramide was determined by a RT-CIM system

As FUT1 and FUT2 mediate the biosynthesis of glycolipid Globo H-ceramide, we explore the possibility that Globo H-ceramide may contribute in part to the observed impacts of FUT1 and FUT2 on cell migration and adhesion. As expected, FUT1 or FUT2 silenced T47D and FUT1 silenced MCF7 tumors expressed decreased Globo H by immunohistochemical staining and flow cytometry with anti-Globo H antibody (Fig. 6a). Exogenous addition of Globo H-ceramide increased cell surface expression of Globo H to similar levels in T47D and MCF7 cells transfected with shLuc, shFUT1, or shFUT2 (Fig. 6b). Exogenous addition of Globo H-ceramide promoted cell adhesion in T47D cells expressing shLuc, but could not ameliorate the inhibitory effect of FUT2 knockdown on cell adhesion (Fig. 6c). On the other hand, the addition of Globo H-ceramide significantly increased transwell migration of MCF7 and partially rescued the inhibition of migration mediated by FUT1 knockdown (Fig. 6d). These results suggest that cell migration and adhesion regulated by FUT1 and FUT2 is in part contributed by Globo H-ceramide, but also involves glycoproteins containing alpha 1, 2-linked fucose.

## Discussion

We have demonstrated the important roles of FUT1 and FUT2 in breast cancer as evidenced by their regulation of cell morphology, proliferation, adhesion, migration, and mammosphere formation in vitro and tumorigenicity and metastasis in vivo. Exogenous addition of Globo H-ceramide, a glycan product of FUT1 and FUT2, partially rescued shFUT1 reduced migration, but not shFUT2 reduced cell adhesion.

Our findings are consistent with previous reports that FUT1 increased alpha 1, 2-fucose of cell surface to promote tumorigenesis and metastasis in colon cancer^12,13^, and that FUT1 served as a promoter for cancer progression in ovarian, hepatocellular, and oral cancer^21–23,33^. The proliferation of hepatocellular carcinoma enhanced by Hepatitis B virus X protein was attributed to downregulation of microRNA-15b which resulted in upregulation of FUT2, leading to FUT2-induced Globo H expression^34^. The involvement of FUT1 and FUT2 in angiogenesis has also been elaborated^22,35^. In light of the report of growth promotion by FUT1 in HER2-overexpressing breast cancer^26^, our data in HER2 negative breast cancer indicated that FUT1 mediated alpha 1, 2-linked fucose plays a crucial role in breast cancer progression regardless of HER2 status.

Many glycan products of glycotransferases are known to contribute to malignant tumor transformation in cancer. Enhanced expression of glycotransferase such as MGAT5 (mannoside acetyl glucosaminyl transferase 5) that makes beta 1, 6-GlcNAc-containing N-glycans is correlated with poor prognosis in breast and colorectal carcinomas^36,37^. Suppression of EMT, cell motility, tumor formation and metastasis were observed in MGAT5-/- mice lacking beta 1, 6-GlcNAc-containing glycans^38^. To date, FUT1 and its glycan products are relatively better studied than FUT2 in relation to cancers. For example, Le^y^ antigen expression has been found at high levels in a variety of human epithelial carcinomas. Suppression of FUT1 with reduced Le^y^ expression inhibited epidermoid carcinoma (A431) and HER2-overexpressing breast cancer (NCI-N87) via EGFR^19,26^, and decreased adhesion of dendritic cells over endothelial cells via reduced Le^y^ on ICAM-2^39^. In this study, the evidence linking Le^y^ to various FUT1 related function was inferential, however, we showed the contribution of Globo H-ceramide, a glycan product of FUT1 and FUT2, to some of the phenotypes attributable to FUT1/FUT2. Indeed, FUT2-induced Globo H has been found to be increased by Hepatitis B virus X protein to enhance hepatocellular carcinoma proliferation^34^.

Smad3 null mice, a spontaneous colorectal model, expressed abnormal pattern of alpha 1, 2-fucosylated glycans with extremely high level of FUT2 mRNA, but displayed no differences in the number and size of colorectal tumors regardless of the FUT2 genotype^40^. In contrast, our study showed that FUT2 knockdown significantly abrogated cellular proliferation, adhesion, tumor formation in T47D, which implied a tissue-specific role of FUT2 in different tissues/cells. In addition to their functional redundancy, FUT1 and FUT2 may mediate alpha 1, 2-fucosylation of different glycoproteins as implied by Globo H-mediated reversal of FUT1 knockdown reduced motility but not FUT2 knockdown decreased adhesion. Consistent with this, previous studies showed that FGA1 is lost from pancreas in FUT1-null mice, whereas, both FGA1 and FGM1 are completely absent in antrum, cecum, and colon in FUT2-null mice^9^.

FUT1 and FUT2 null mice display reduced expression of FGM1 and FGA1, but no gross phenotypic abnormalities^8,41^. FUT1 and FUT2 are responsible for alpha 1, 2-fucosyltransferase, since a third alpha 1, 2-fucosyltransferase gene called Sec1 is a pseudogene which is inactivated by insertion of a nucleotide resulting in a frameshift in the coding sequence^42^. Inhibition of cancer cell progression, angiogenesis and metastasis by FUT1 or FUT2 knockdown reduced expression of alpha 1, 2-fucosyl glycans has been found in many cancers including breast cancer. Besides, breast stromal cells express low level of FUT1 and absent FUT2. Our and other studies both demonstrate that FUT1 and FUT2 play crucial roles in more than one subtype of breast cancer, at least including HER2-overexpressing and HER2 negative^26^. Thus FUT1 and FUT2 may serve as good drug targets for breast cancer therapy.

## Materials and methods

### Cell lines

The human embryonic kidney cell line HEK293T and Breast cancer cell lines T47D, MCF7, and MDA-MB-231 were obtained from American Type Culture Collection. HEK293T, T47D and MDA-MB-231 were cultured in Dulbecco’s Modified Eagle Medium (DMEM) with 10% fetal bovine serum (FBS). MCF7 were cultured in Minimum Essential Media (MEM) with 10% FBS.

### Lentivirus Production and Transduction

The lentiviral constructs pLKO.1-puro harboring FUT1-specific shRNA (TRCN0000036078 and TRCN0000036074), FUT2-specific shRNA (TRCN0000036102, TRCN0000431129, and TRCN0000418672), or shLuc (TRCN0000231715) were obtained from the National RNAi Core Facility (Institute of Molecular Biology/Genomic Research Center, Academia Sinica, Taiwan). The VSV-G-pseudotyped lentiviruses were produced by co-transfecting lentiviral-based expression vector pLKO.1-puro harboring a specific shRNA encoding sequence, packaging plasmid pCMV-R8.91, and pMD.G into HEK293T cells. Infectious lentiviruses were harvested at 72 h after transfection and were concentrated by ultracentrifugation (25,000 rpm, 90 min, 4 °C). T47D and MCF7 cells were plated at 5 × 10^5^ cells per well in six-well plates and transduced with lentivirus in the presence of 8 μg/ml polybrene (Sigma). The transduced T47D and MCF7 cells were replaced with selective medium containing 0.5 μg/ml and 0.25 μg/ml puromycin (Sigma), respectively, at 72 h post-transduction. The stable cells were still selected more than 5 day until the 90% death of control cells which were not transduced with lentivirus.

### Plasmid DNAs and Transfection

MCF7 and T47D genomic DNA were used as templates for FUT1 and FUT2 in a PCR, respectively. Flag-tagged FUT1 was amplified by a primer pair of Flag-FUT1-FW (AAGAATTCACCATGGACTACAAAGACGATGACGACAAGATGTGGCTCCGG AGCC) and FUT1-REV (AAGAATTCTCAAGGCTTAGCCAATGTCCAGAGTGGAGAC). Flag-tagged FUT2 was amplified by a primer pair of Flag-FUT2-FW (AAGAATTCACCATGGACTACAAAGACGATGACGACAAGATGCTGGTCGTTCAGATGCCTTTC) and FUT2-REV (AAGAATTCTTAGTGCTTGAGTAAGGGGGACAGG). The amplified Flag-FUT1 and Flag-FUT2 cDNA were inserted into EcoR I site of pcDNA3.1 (Invitrogen). All transfection was performed using electroporation. The condition of MCF7 was 900 voltage (V), 30 m-second (ms), 2 pulse (P); T47D was 1400 V, 30 ms, 1 P; MDA-MB-231 was 1400 V, 10 ms, 3 P. The ratio of cells to recombinant DNA is 1 × 10^6^ cells to 10 μg of DNA. 3 × 10^6^ cells were plated in 100 mm Petri-dish. The cells were harvested for subsequent analyses at 48 h post-transduction.

### Quantitative real-time-PCR

Total RNA was extracted and reverse-transcripted to cDNA with oligo(dT) primer. RT-PCR for simultaneous detection and quantification of the cDNA samples was performed on an ABI Prism 7300 Sequence Detection System and analyzed with the ABI Prism 7300 SDS software (Applied Biosystems). SYBR Green I detection (Applied Biosystems) was used to detect FUT1 and FUT2 gene expression. The primers were as follows: FUT1: CCGGTTTGGTAATCAGATGG and CTCAAGTCCGCGTACTCCTC as well as FUT2: ATCATGACCATTGGGACGTT and GTGCTTGAGTAAGGGGGACA from previous study^43^. The cDNA sample were used for qPCR reaction as 50 °C for 2 min, 95 °C for 10 min, followed by 40 cycles of 95 °C for 10 s and 60 °C for 1 min. The end-point used in the real-time quantification was calculated by the ABI Prism 7300 SDS software, and the threshold cycle number (Ct value) for each analyzed sample was calculated. GAPDH was used as the internal control in SYBR Green system.

### Flow Cytometry

Aliquots of cells (1 × 10^5^) were stained with anti-Globo H-Alexa488 (Vk9)^10^, or anti-CD24-PE and anti-CD44-APC antibodies at 4 °C for 1 h. All analyses were done with a FACSCanto flow cytometer using the CellQuest program (BD Biosciences).

### Wound healing motility assay

Cells were plated on 6-well plates until confluent, and then were starved for overnight. The cell monolayer was scratched with a linear scratch by a sterile 200 μl pipette tip. After scratching, cell monolayer was washed once with PBS then cultivated with 5% FBS containing medium. The wounded areas were photographed at 0 and 48 h after scratching. The wounded areas at 0 h and remain area at 48 h were calculated by image J software.

### Cell adhesion assay

Cell adhesion was assessed using RT-CES apparatus (Real Time Cell Electronic Sensing, ACEABIO). ACEA’s 96 microtiter plates were coated with the appropriate dilution in PBS of fibronectin (25 μg/ml, Sigma), type IV collagen (2 μg/ml, BD biosciences), or laminin (5 μg/ml, Sigma) at 37 °C for 1 h and then were blocked with 1% BSA for 1 h at 37 °C. Cells were seeded at 2.5 × 10^4^ per 100 μl of culture medium in ACEA’s 96 microtiter plates. Cell adhesion was continuously monitored every 10 min using the RT-CES for a period of 1 h. For the exogenous addition of Globo H-ceramide, cells were incubated with 50 μg/ml Globo H-ceramide in serum-free medium for overnight.

### Cell migration

Cell migration was measured using a RT-CIM apparatus (ACEABIO). Cells were starved overnight in serum-free medium, and then seeded at 5 × 10^4^ per 100 μl with serum-free medium in the upper chamber with 8 μM pore size. The lower chamber was contained 10% serum medium. Cell migration was continuously monitored every 2 h by ACEA’s 16 microtiter plates for a period of 0 to 18 h.

### Cell invasion

The 50 μl of matrigel was prepared in chill serum-free medium in a concentration of 1 mg/ml and placed onto the upper chamber of transwell (24-well, Corning). After incubation at 37 °C for 1 h, 10% FBS (v/v) was supplemented in medium and placed in the lower chamber as a chemoattractant. Then, 1 × 10^5^ cells in serum-free medium were added to the upper chamber and incubated at 37 °C for 24 h. For quantification, cells were fixed with 4% formalin, and subsequently stained with 0.2% crystal violet. After the upper side of the membrane was gently wiped, at least five fields of invading side were imaged and the attached cells were counted.

### Cell proliferation assay

Cells were seeded at 2.5 × 10^3^ cells per well on 96-well plates (Corning). At 24, 48, 72, and 96 h after cells seeded, alamar blue (BioRad) was added to a final concentration of 1/10 dilution and cells were harvested at 37 °C, 5% CO_2_ for 3 h. The absorbance was measured at 544/590 nm and determined by SpectraMax M2 Reader. Assays were performed in triplicate.

### Mammosphere formation assay

Single cell suspensions of cell lines were suspended at a density of 1,000 cells/ 150 μl in DMEM/F12 containing 0.4% BSA, 10 ng/ml EGF, 20 ng/ml bFGF, 1 μM hydrocortisone, and 1x B27 supplement and then were seeded on ultra low attachment plates (Corning). Cultures were fed weekly over than two weeks.

### Tumorigenicity assays

Six-week-old NOD/SCID mice were purchased from National Laboratory Animal Center, Taiwan. T47D (5 × 10^6^) or MCF7 cells (1 × 10^7^) expressing shLuc, shFUT1, or shFUT2 in 0.1 ml of 50% Matrigel (BD Biosciences) plus 50% supplemented RPMI-1640 were injected subcutaneously into mammary fat pad of 8-week-old, female mice. The tumor volume was regularly monitored by measuring the length (*l*) and width (*w*) and calculating the volume (*V* = *π* / 6 × *l* × *w* × [*l* + *w*] / 2). At the end of the experiment, the animals were sacrificed and the tumors were excised and weighed.

### Bioluminescence imaging

The MDA-MB-231 cells were transduced with the lentivirus containing the plasmid pLKO.CMV.luc.GFP. GFP + cells at 95% purity sorted by FACS were then transfected with empty vector pcDNA3.1, Flag-FUT1 or Flag-FUT2 containing vectors followed by selection with 1 mg/ml G418 (Promega). The cells (3 × 10^6^) were injected intravenously through the tail vein of the NOD/SCID mice (*n* = 3 in each group, power = 0.80, *α* = 0.05) and three individual experiments were carried out. The substrate luciferin (Nanolight) was injected into the intraperitoneal cavity of the mice at 75 mg/kg (15 mg/ml luciferin), ~5 minutes before imaging. Mice were anesthetized with 2.5% isoflurane/oxygen and placed on the imaging stage. Ventral and dorsal images were collected for 1 minute using the IVIS (Xenogen). Photons emitted from the lung region were quantified using Living Image software v2.6 (Xenogen). After sacrifice, lungs were excised, fixed in 10% formalin (Sigma, St. Louis, MO, USA) and prepared for standard histopathology evaluation.

### Western Blotting

Cells were incubated in buffer containing 50 mM Tris pH 7.2, 300 mM NaCl, 1% Triton X-100, a panel of protease inhibitor mixture, and 2 mM sodium orthovanadate for 20 min. Whole cell lysates were subjected to NuPAGE (10% Bis-Tris gels, Invitrogen) and Western blotting with anti-E-cadherin antibody (BD Biosciences), anti-twist antibody (Santa Cruz), anti-fibronectin antibody (Millipore), anti-vimentin antibody (Abcam, 5G3F10), and anti-tubulin (Sigma). The immunoblots were developed with the ECF system (GE Healthcare).

### Immunohistochemistry

T47D and MCF7 tumor xenografts from NOD/SCID mice were fixed in 10% phosphate-buffered formalin and embedded in paraffin. Paraffin sections were cut at a thickness of 2 μm, mounted on SuperFrostTM Plus microscopy slides (Menzel-Gläser), and dried at 70 °C for 2 h. The sections were dewaxed in xylene and rehydrated according to the standard histopathological procedures. The slides were first placed in the solution of Tris-EDTA pH 9.0 and autoclaved for 7.5 min for antigen retrieval. The slides were then blocked with 3% hydrogen peroxide for 15 min and incubated with biotin conjugated UEA-1 lectin (Vector Laboratories) for 1 h at room temperature followed by fluorescein-conjugated streptavidin (Jackson ImmunoResearch) for 1 h at room temperature. Finally, the slides were stained with Hoechst 33258 for 15 min and mounted with Vectashield (Vector Laboratories). For Globo H staining, the slides were stained using Benchmark XT machine (Ventana). The slides were added with anti-Globo H antibody (Vk9) in power block HK085-5K solution (Ventana) at 37 °C for 1 h followed by biotin conjugated anti-IgG and streptavidin-HRP for 20 min and applied with iVIEW DAB Detection kit (Ventana). Leica autostainer XL (CV5030) machine was then used for counter staining with Mayer’s hematoxylin and mounting. The images were collected with Leica automatic upright microscope (DM 6000B).

### Statistical Analysis

All experiments were repeated at least three times. The data were analyzed using the two-tailed Student’s t test or one-way ANOVA, depending on number of groups compared. When one-way ANOVA was taken, we performed Tukey’s range test as post hoc test to analyze the variances. For both methods, a *P* value of 0.05 or less was considered significant. The statistical analyses were performed with Prism 5.0 (GraphPad Software, La Jolla, CA, USA)

## Supplementary information

Suppl-Figure 1

Suppl-Figure 2

Supplementary figure legends
