# Supplementary figures and images for: Fucosyltransferase 1 and 2 play pivotal roles in breast cancer cells
Source: Cell Death Discov. 2019 Mar 6;5:74. doi: 10.1038/s41420-019-0145-y (PMC6403244; doi:10.1038/s41420-019-0145-y)

**a****T47D****Relative fold change in mRNA**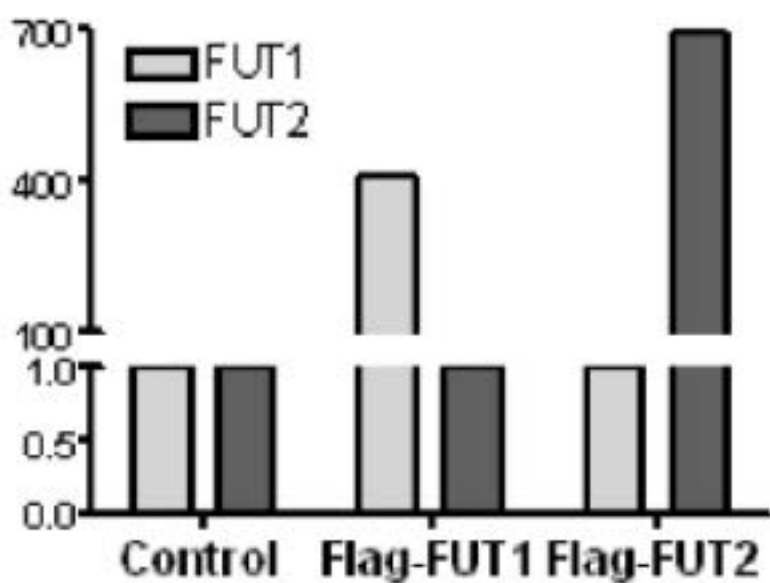**b****MCF7****Relative fold change in mRNA**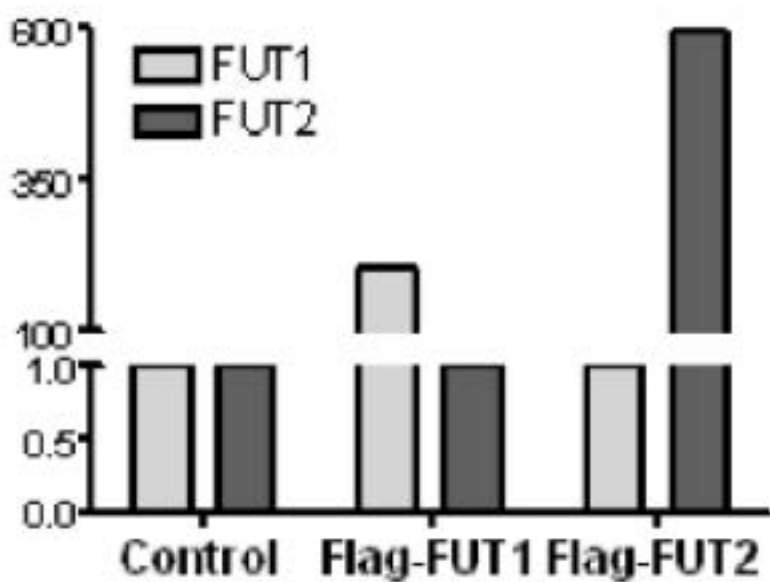

Supplement: Supplementary file 1 — Suppl-Figure 1 [file 41420_2019_145_MOESM1_ESM.pdf]

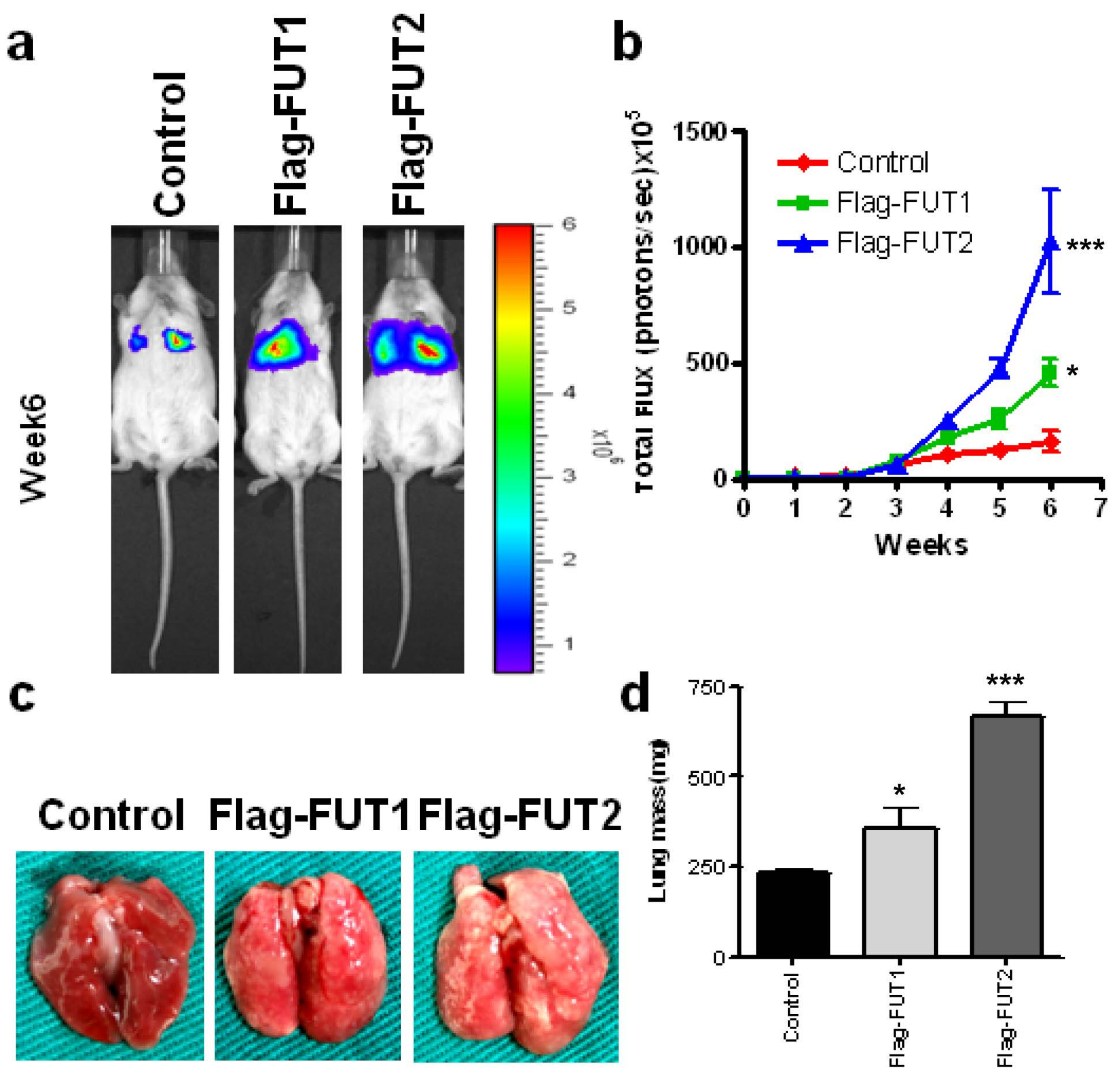

Supplement: Supplementary file 2 — Suppl-Figure 2 [file 41420_2019_145_MOESM2_ESM.pdf]
