# Supplementary material for: Fucosyltransferase 1 and 2 play pivotal roles in breast cancer cells
Source: Cell Death Discov. 2019 Mar 6;5:74. doi: 10.1038/s41420-019-0145-y (PMC6403244; doi:10.1038/s41420-019-0145-y)
Supplement: Supplementary file 3 — Supplementary figure legends [file 41420_2019_145_MOESM3_ESM.docx]

**Supplementary Figure 1 FUT1 or FUT2 overexpression increases the mRNA　levels of FUT1 or FUT2.** **a,** T47D and **b,** MCF7 cells expressing vector control, Flag-FUT1, or Flag-FUT2 were analyzed for FUT1 or FUT2 mRNA expression..

**Supplementary Figure 2 FUT1 and FUT2 are responsible for metastasis. a,** The experimental metastatic ability of bioluminescent MDA-MB-231 expressing vector control, Flag-FUT1, or Flag-FUT2 was determined by quantitative photon counts emitted from the lung region weekly. **b,** Bioluminescent images of metastases as well as **c,** display of lung organs and **d**, weight of lung tumor mass at week 6 after intravenous injection of indicated cells into NOD-SCID mice. Similar results were obtained in 2 individual experiments with 3 mice in each group. **p* <0.05; ***p* <0.01; ****p* <0.0001
